# A preclinical setup for spatially fractionated radiation therapy with electrons

**DOI:** 10.1002/mp.70448

**Published:** 2026-04-24

**Authors:** Edward R J F Taylor, Jia‐Ling Ruan, Salomé Paillas, Iain D C Tullis, Geoff S Higgins, Kristoffer Petersson

**Affiliations:** ^1^ Department of Oncology University of Oxford Oxford UK

**Keywords:** electron, FLASH, mini‐grid, preclinical, spatial fractionation, subcutaneous tumor

## Abstract

**Background:**

Spatially fractionated radiation therapy (SFRT) has therapeutic potential as a priming therapy which boosts tumor control. However, the optimal delivery and spatial fractionation parameters have not been deciphered and the mechanisms at play are not yet fully understood.

**Purpose:**

This paper highlights our preclinical setups for mini‐grid SFRT with 6 MeV electrons delivered at conventional to ultrahigh dose rates, using a flexible collimator system. These setups let us explore relevant spatial fractionation parameters to observe their effect on tumor growth and normal tissue toxicity. Preclinical studies here may reveal the parameters of highest clinical relevance for SFRT and combination therapies.

**Methods:**

For preclinical experiments with electron spatial fractionation, 6.5 mm thick brass collimators were made with 7‐ (or 19‐) hole hexagonally packed ∅ 0.65‐2 mm apertures, with 1.6‐5 mm CTC distances. Irradiated EBT‐XD Gafchromic film downstream of collimators were analyzed to obtain peak‐to‐valley dose ratios (PVDR), full width at half maxima (FWHM), and peak doses at various depths in solid water, and at surface when increasing the separations from collimators in air. Male and female C57BL/6 mice were injected subcutaneously with UPPL1541 bladder cancer cells in the right flank. After 10–13 days, a single dose treatment was delivered to the tumors with either ∅14 mm circular homogeneous field (10 Gy delivered at 3 kGy s^−1^), or using a 7‐hole (∅ of 2 and 5 mm center‐to‐center distances) spatially fractionated field; peak doses of 30 Gy delivered at 820 Gy s^−1^, 20 Gy at 860 Gy s^−1^, and 20 Gy at < 0.1 Gy s^−1^. Tumor growth and time to triple tumor volume (TTTV) were measured and compared between treatment regimens.

**Results:**

Similar PVDRs were obtained with 7‐ and 19‐hole inserts (35 and 31 at surface, respectively). Peak widths increased with depth, and maximal peak dose rates were > 1.8 kGy s^−1^. A displacement in air from the collimator exit decreased PVDRs at the phantom surface; from 32 to 16 at ∼10 mm distance, and to 6 at ∼20 mm distance. Peak doses also reduced to ∼57 % at 10 mm distance, and to ∼33% at 20 mm distance. Film measurements at the mouse phantom surface produced peak and valley dose rates of > 850 Gy s^−1^ and ∼60 Gy s^−1^ respectively, with a PVDR > 14. Tumor growth delays for spatially fractionated FLASH 30 Gy (peak dose, with a 2.1 Gy valley dose, and a 10 Gy average dose) and homogeneous FLASH 10 Gy electron irradiation regimens were similar. Both regimens also demonstrated significantly longer TTTV compared to control and spatially fractionated conventional 20 Gy (peak dose, with a 1.4 Gy valley dose, and a 6.7 Gy average dose) regimens (*p* < 0.05). No significant differences in body weight and skin damage were observed, indicating acceptable treatment tolerability.

**Conclusions:**

Spatially fractionated electron FLASH treatments with 30 Gy peak doses and 2.1 Gy valley doses provide effective tumor growth delay and prolonged tumor control akin to 10 Gy homogeneous irradiations. Here we demonstrate that combining spatial modulation, higher peak doses, and FLASH dose rates can produce favorable tumor response.

## 1 INTRODUCTION

Spatially fractionated radiotherapy (SFRT) represents a step change from traditional approaches to radiotherapy. Instead of homogeneous distribution of dose throughout tumor sites, with a lethal dose to every tumor cell, this technique aims to deliver heterogenous geometric patterns of high (peak) and low (valley) dose regions. Instead of killing tumor cells with irradiation alone, the rationale behind this irradiation method is to kickstart antitumor immunological response, and exploit the benefits mediated by abscopal, bystander effects, vascular damage, perfusion, inflammatory and immunological mechanisms that occur because of heterogeneous dose delivery.^1^, ^2^, ^3^

The approach may lower normal tissue toxicity, potentially enhance tumor control and thereby widen the therapeutic window, using small volume regions of higher peak doses to harness greater normal tissue tolerance by reducing deleterious dose‐volume effects. Treatments using SFRT with high doses have potential to better target bulky (> 5 cm), and radioresistant tumors, especially those close to sensitive structures and organs at risk (OAR).^4^, ^5^

SFRT techniques are classified by the spatial scale of their beam modulation. Microbeam radiotherapy (MRT) use beam widths on the micrometer scale (< 100 µm), showing great biological benefits compared to homogeneous irradiation but require synchrotron‐generated X‐rays and offer limited clinical applicability due to infrastructure and dosimetry challenges.^2^, ^6^, ^7^, ^8^, ^9^ Minibeam radiotherapy (MBRT) employ sub‐millimeter beam widths (100 µm‐1 mm), which have been delivered by X‐ray tube based systems and proton beams in preclinical experiments, but would also be challenging to translate clinically.^2^ GRID therapy, delivered with centimeter‑scale holes in a static collimator, has been translated clinically but is fundamentally two‑dimensional. Lattice radiotherapy (LRT) extends GRID into three dimensions by placing multiple spherical high‑dose vertices inside the tumor, typically 15–25 Gy each, while maintaining a high peak‑to‑valley dose ratio (PVDR) throughout both normal tissue and tumor volumes.^2^, ^10^, ^11^, ^12^, ^13^ LRT delivered with volumetric‑modulated arc therapy (VMAT) or helical tomotherapy has produced encouraging responses in gynaecological, lung, head‑and‑neck, and soft‑tissue sarcoma cases with limited toxicity.^14^, ^15^, ^16^, ^17^, ^18^

Despite these early successes, clinical adoption remains slow because fundamental treatment‑planning questions are unresolved. Most protocols prescribe only the vertex (peak) dose, ignoring potentially influential parameters such as valley dose, PVDR, fractionation scheme, sequencing, or vertex geometry.^19^, ^20^, ^21^, ^22^ Similarly, links between treatment efficacy and a given parameter have been complicated by inconsistent primary endpoints, prior or post conventional irradiation or fractionation,^23^ or combining treatments with chemotherapy or immunotherapy.^18^, ^24^, ^25^, ^26^, ^27^ Optimal vertex number, size, and spacing, and how these should be adjusted to tumor composition or proximity to OARs are unknown.^5^, ^6^ Without consistent standards, comparisons across studies are difficult and mechanistic insights remain elusive.^28^

Progress therefore would benefit from additional reproducible pre‑clinical platforms that can isolate dosimetric variables and correlate them with biological endpoints in vivo. Mega‑electron‑volt (MeV) electron beams are well‑suited to this task. They can deliver high dose rates through simple scanning or collimation, and their radiobiological effectiveness is comparable to that of megavoltage (MV) photons for the small field sizes and doses typical of SFRT.^29^, ^30^ Moreover, electrons lend themselves naturally to ultra‑high dose rate (FLASH) delivery, which has been shown to spare normal tissue while preserving tumor control in conventional field geometries. Combining FLASH with SFRT could further mitigate motion‑induced blurring of steep dose gradients and enhance normal‑tissue protection.

Here we present a preclinical SFRT platform based on an electron linear accelerator (linac) that can deliver 6 MeV electron beams with dose rates ranging from conventional (CONV) to ultrahigh (FLASH), combined with a flexible collimator system designed to generate customizable spatial dose patterns suitable for small animal models. Our first in‐vivo results highlight that 30 Gy (peak dose with 2.1 Gy valley dose and an average dose of 10 Gy) SFRT can achieve similar tumor response as 10 Gy homogeneous field radiotherapy (RT). This platform establishes a flexible experimental framework (we present data for 10 different collimators) to assess how parameters such as peak dose, valley dose, PVDR, and fractionation define the therapeutic benefit of SFRT. Ultimately, insights gained with this platform will inform rational, standardized treatment protocols for future clinical translation.^31^, ^32^ Our electron SFRT (eSFRT) arrangements also enable the combination of ultrahigh dose rate FLASH and SFRT to be explored.

## 2 METHODS

### 2.1 Linac

Experiments were conducted using our preclinical FLASH‐optimized in‐house 6 MeV nominal electron linac. The accelerator employs an Elekta SL75 travelling wave waveguide with an S‐band radiofrequency magnetron (Teledyne e2v‐M5125 type). Macropulse widths are 3.4 µs, with peak currents of 100 mA, a duty cycle of 0.1%, and repetition rates up to 300 Hz. Beam energy can be monitored from pulse‐to‐pulse, ^33^whilst a non‐intercepting electron beam charge monitor (toroidal inductive sensor) can be used to precisely control the delivery by accumulating charge. ^34^A circular beam of ∼5 mm diameter full width at half maximum (FWHM) is produced, which emerges from the beryllium copper exit window. ^33^, ^35^ A Ti scattering foil (30 µm thick, positioned 8.5 mm downstream from the output window) is used to spread out the beam, and 6 mm thick brass plates with various apertures are used to collimate the beam immediately upstream of the (mouse/cell) sample position.

### 2.2 Mini‐grid SFRT setup

**FIGURE 1:**
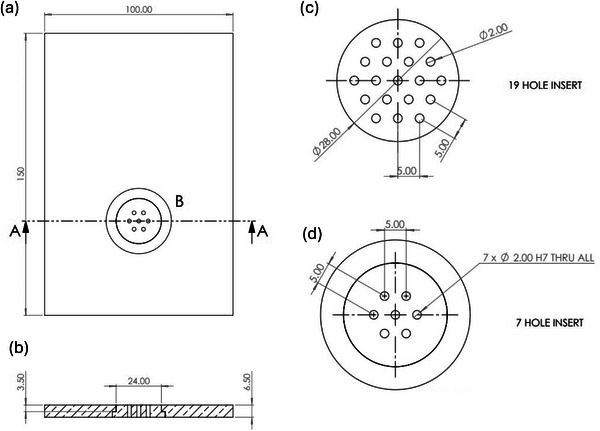
Electron mini‐grid SFRT brass inserts with 7 and 19 holes, with hole diameters of 2 mm, and CTC distances of 5 mm, hexagonally packed and placed within a brass collimator plate (all dimensions are displayed in mm): (a) Beam's eye view of 7‐hole insert within brass backer plate (b). Top view of 7‐hole insert within brass backer plate. (c) 19‐hole brass insert dimensions. (d) 7‐hole brass insert dimensions.

For our mini‐grid SFRT arrangements, 7‐hole, and a 19‐hole hexagonally packed 6.5 mm thick brass inserts were manufactured (Figure 1). These coin shaped inserts were designed to fit tightly within the 6.5 mm, 100 mm x 150 mm, brass collimator plate positioned adjacent and downstream of the energy monitor. The insert used for our pilot mice experiment was manufactured with 2 mm holes, and a CTC distance of 5 mm (Figure 1d). Eight additional 7‐hole inserts were also manufactured with smaller hole diameters and CTC distances (supplementary Figures S1‐S2). The source‐to‐surface distance (SSD) for electron irradiations was set to 72 cm, with the collimator housing and brass collimator plate adjacent and directly upstream of the sample position.

### 2.3 Dosimetry

To obtain dose, dose rate, and PVDR measurements, 34 × 34 mm^2^, Gafchromic EBT‐XD films (Ashland Inc., Covington, KY, USA) were used. At 24 hours postirradiation films were read out using a film scanner (Epson Perfection v850 Pro, Seiko Epson Corporation, Nagano, Japan) at 300 dpi. Films were analyzed (red channel) using ImageJ (v2.14.0/1.54f) and had previously been calibrated with 6 MeV electron clinical beam from a Varian TrueBeam linac (Varian Medical Systems Inc., Palo Alto, CA, USA) at the [removed for peer review]. For each measurement, at least 5 cm of solid water (150 × 150 mm^2^ RW3 slabs of 1‐, 2‐, 5‐, and 10‐mm thicknesses from PTW‐Freiburg), was used as backscatter material. Two unirradiated background films were also used for the conversion of optical density to dose. The overall uncertainty in dosimetry was estimated to be 4% (including a measured output variation of within 2%).

**FIGURE 2:**
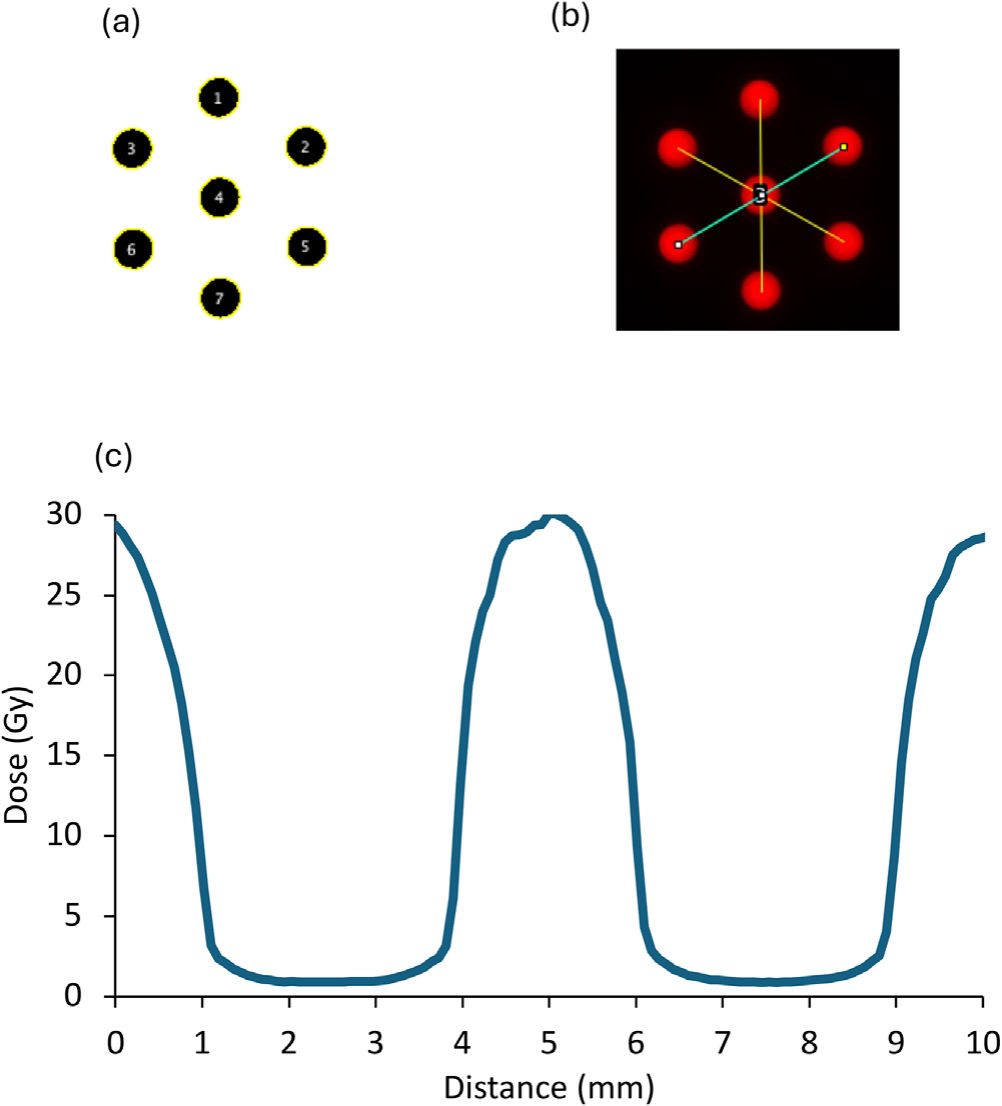
(a) Peaks detected (in black) algorithmically using imageJ (v2.14.0/1.54f) from an example EBT‐XD film dose measurements using the 7‐hole electron collimator (Figure 1d). (b) Three single‐pixel lines separated rotationally by 60^∘^ were analysed (with profiles in yellow and blue, and peaks in red); with each line connecting the centers of adjacent peaks along the principal axes of symmetry that intersected the CAX. (c) Dose across the blue single‐pixel line profile shown in (b).

Macro scripts were developed to obtain dose values in every pixel on the scanned films. Three single‐pixel lines separated rotationally by 60^∘^ were analysed; with each line connecting the centers of adjacent dose peaks along the principal axes of symmetry that intersected the center axis (CAX) (Figure 2). From dose profiles along these lines, average values of FWHM, dose maxima and minima were obtained − where the ratio of these was used to extract an average PVDR value.

## 3 PRECLINICAL BIOLOGICAL METHODS

### 3.1 Animal experiments

All animal work was performed in accordance with UK Home Office Guidelines, following the ARRIVE (Animal Research: Reporting of* In Vivo* Experiments) guidelines, ^36^ and approved by the [removed for peer review] Animal Welfare and Ethical Review Body (AWERB), under [removed for peer review] project licence [removed for peer review]. All male and female C57BL/6J mice (aged 8–10 weeks) were purchased from Charles Rivers UK Ltd. Mice were housed in groups of three to four in individually ventilated cages in a temperature‐controlled environment with a 12‐hours reversed‐phase light/dark cycle (lights on 07:00 h) and provided with food and water ad libitum at Department of Biomedical Services, Radiobiology Research Institute, [removed for peer review].

### 3.2 Cell culture

UPPL1541 bladder carcinoma cells were sourced from applied biological materials (ABM), used within 5 passages after purchase, and confirmed to be free of mycoplasma contamination. Cells were maintained in high‐glucose DMEM (Gibco, 11995065) supplemented with 10% fetal bovine serum (FBS) and 1% penicillin‐streptomycin (100 U/mL). Cultures were maintained at 37°C in a humidified atmosphere containing 5% CO_2_. For in vivo injections, cells were mixed 1:1 with high‐concentration Matrigel (Corning, 354262).

### 3.3 Subcutaneous tumor model

**FIGURE 3:**
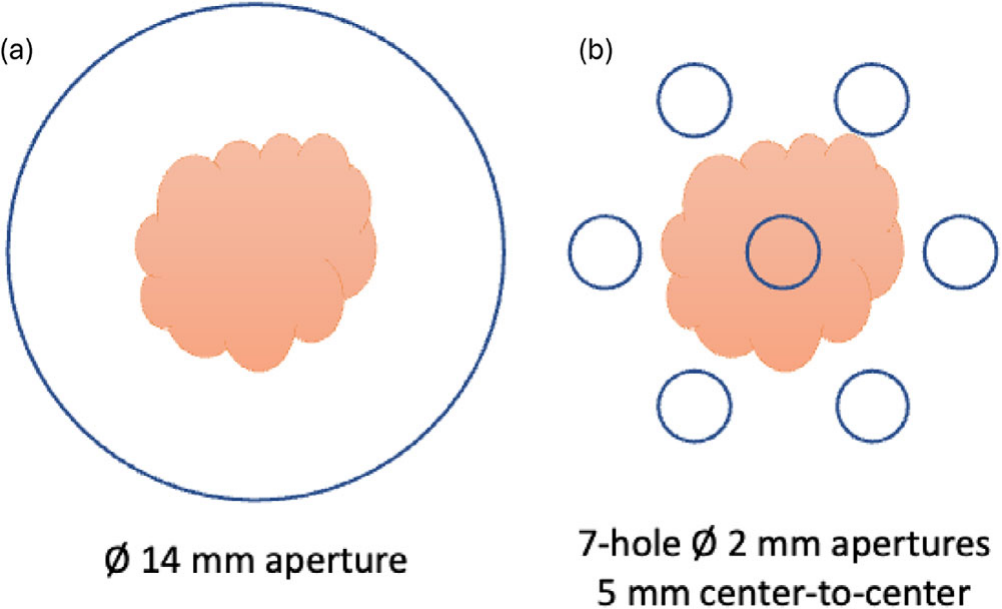
Illustration of how the tumor (schematic representation of a tumor at ∼100 mm^3^ volume, ∼7‐mm diameter) was aligned with (a) the center of the 14‐mm diameter aperture collimator for homogenous RT, or (b) with the central aperture of the 7‐hole 2 mm diameter apertures (5 mm center‐to‐center distance) collimator for mini‐grid SFRT.

A total of 31 C57BL/6J mice (mean weight 26.04 ± 1.03 g) were injected subcutaneously in the right flank with 5 × 10^6^ UPPL1541 cells. Treatments (Table 1) were performed in two experimental batches (day 10 and day 13 post‐inoculation), with animals randomly distributed across all treatment groups within each batch and treated at comparable tumour volumes (90.83 ± 21.78 mm^3^, mean ± SD; CI 82.84–98.83 mm^3^; average tumour thickness ≈3 mm; individual baseline values shown in Figure S5) to minimize variability due to tumour growth stage. For the radiotherapy, the mice were anesthetized using isoflurane (4% for anaesthetic induction and 2% for maintenance, with total anaesthesia time of less than 10 minutes), supplemented with 95% oxygen (1/1 mixture with air resulting in a mixture of approximate 60% oxygen), and then placed upright in a mouse cradle in front of the horizontal beam.^37^ Beam collimation was achieved using a 6.5 mm thick brass plate, with 7 hexagonally packed apertures of 2 mm diameter with a center‐to‐center (CTC) distance of 5 mm (as seen in Figure 1d) for an SFRT treatment, or a single 14 mm diameter aperture for homogenous field irradiations. The mice were positioned such that the collimator shielded everything other than the subcutaneous tumor which was aligned with the center hole of the SFRT collimator, or to the center of the 14 mm diameter aperture (Figure 3).

**TABLE 1:** Electron mice irradiation regimens.

Regimen label	Brass collimation / hexagonally packed spatial fractionation	Dose prescription	Single dose delivered (Gy)	Peak dose rate (Gy s^−1^)	Peak dose‐per‐pulse (Gy)	Mice in cohort (*n)*
eFLASH 10 Gy	∅14 mm aperture	Homogeneous	10	3,000	5	7
eSFRT‐CONV 20 Gy	7‐hole insert, ∅2 mm, 5 mm ctc	Peak, Valley, Average	20, 1.4, 6.7	<0.1	<0.004	7
eSFRT‐FLASH 20 Gy	7‐hole insert, ∅2 mm, 5 mm ctc	Peak, Valley, Average	20, 1.4, 6.7	860	2.5	6
eSFRT‐FLASH 30 Gy	7‐hole insert, ∅2 mm, 5 mm ctc	Peak, Valley, Average	30, 2.1, 10	820	2.5	7

Tumor size was monitored using callipers, and animals were euthanized once tumor size exceeded 700 mm^3^. Tumor volume was calculated using the formula:

(1) $$\mathrm{Volume}=\mathrm{Length}\times \mathrm{Width}\times \mathrm{Height}\times \frac{\pi }{6}$$

**FIGURE 4:**
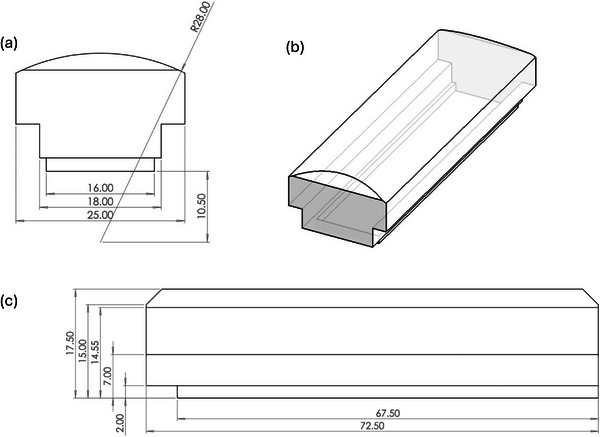
Mouse phantom used for dose delivery prescription and validation using GafChromic EBT‐XD film at the front curved surface of the phantom, facing the beam when placed in mouse cradle. All dimensions are displayed in mm: (a) Top view, (b) Isometric view, (c) Side view.

Prescribed surface peak doses were verified with GafChromic EBT‐XD film measurements before and after mice irradiation, at the surface of a mouse phantom (Figure 4), positioned as the mice in the mouse cradle in the beam (Figure S1). Average doses were analysed over a 6.6 × 6.6 mm^2^ central area of exposed film (for consistency with measurements for homogeneous irradiations). For the SFRT, the average dose in this area (corresponded to 1/3 of the peak dose, matching the highest SFRT average dose with the homogeneous eFLASH dose of 10 Gy) as well as the peak doses were analysed. Additional online dosimetry was performed with an Advanced Markus ionization chamber (PTW‐Freiburg GmbH, Freiburg, Germany) at 1 mm depth in solid water (15 × 15 × 2.1 cm^3^ RW3 slabs, PTW‐Freiburg GmbH).^38^ The beam energy was maintained for all dose rates by slight detuning of the radiofrequency source and online monitoring of the resulting beam energy.^33^

### 3.4 Statistical analysis

Data were analysed using Excel or PRISM software (GraphPad Prism version 10.5.0, GraphPad Software, Boston, MA, USA). The primary endpoint for in vivo efficacy was time to triple tumour volume (TTTV), defined as the number of days from treatment until the tumour reached three times its individual baseline volume at irradiation. Mice were euthanized when tumours exceeded 700 mm^3^ for humane‐endpoint reasons; this threshold is independent of the primary endpoint, and censoring was applied where appropriate. TTTV was selected a priori because it directly reflects treatment‐dependent tumour growth dynamics and avoids the variability introduced by humane‐endpoint timing, thereby improving sensitivity for detecting differences between regimens.

Kaplan‐Meier curves were constructed using TTTV as the event time and analysed with the Log‐Rank test. Pairwise multiple comparisons were performed with Holm correction for 10 tests. Two‐way repeated measures ANOVA followed by Tukey's HSD test was applied for analyses involving multiple groups. Statistical significance was set at *p* < 0.05.

The study hypothesis was that SFRT would produce a measurable delay in tumor growth relative to untreated controls. Sample size was estimated using historical growth rate variability in the UPPL1541 model with an expected effective size of 20%–30% increase in TTTV. Power calculation (*α* = 0.05, power = 0.8) indicated a minimum of 6–7 animal per group.

## 4 RESULTS

### 4.1 eSFRT

**FIGURE 5:**
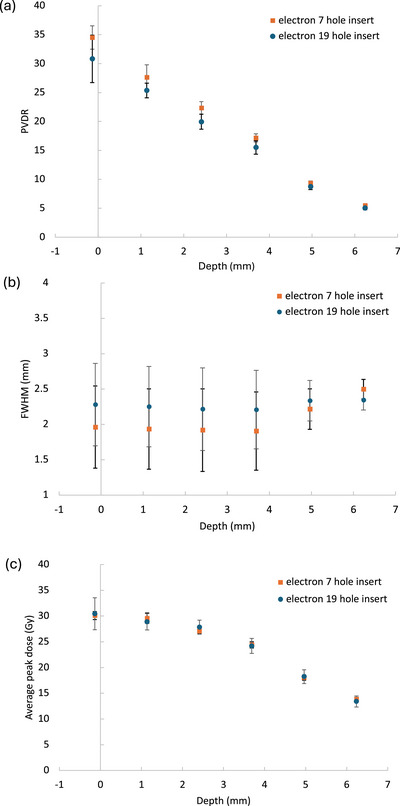
Electron 7‐hole and 19‐hole insert EBT‐XD film irradiations at various depth in solid water where 0 mm indicates the surface. A 5‐pulse delivery was used for both arrangements, here: (a) Average peak‐to‐valley dose ratio (PVDR) with depth. (b) Average FWHM of peaks. (c) Average peak doses. Markers and error bars represent the mean value and standard deviation respectively of values obtained from the films via three symmetric single‐pixel line dosimetric profiles obtained with a 60^∘^ offset from one another whilst intercepting the centre of adjacent peaks, and the central axis.

PVDR values with the 7 and 19‐hole inserts (Figure 1) were similar at 35 ± 2, and 31 ± 4 (Figure 4a). The slightly lesser average PVDR value from the 19‐hole insert was due to lateral off‐axis falloff causing less pronounced peaks in the outer ring of 12 holes. However, differences diminished at increasing depths. The FWHM (Figure 5b) increased with depth for both insert arrangements. The 19‐hole insert showed more FWHM variation, especially at depths > 6 mm, where some peaks began to merge. This was most noticeable for the outer ring of 12 holes with wider FWHMs than those close to the central axis. Peak doses were consistent for both inserts with similar variability (Figure 5, panel c). At surface, peak dose rates were found to be > 1.8 kGy s^−1^. Equivalent simulation and experiments with our eight other collimator designs are highlighted in supplementary materials (Figures S1‐S4).

**FIGURE 6:**
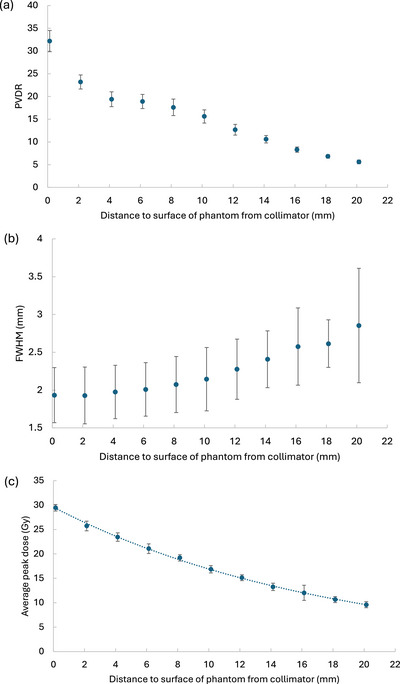
Electron 7‐hole insert EBT‐XD film irradiations, with film placed on the surface of 5 cm thick slab of solid water (15 × 15 × 5 cm^3^). Air separation between the downstream surface of the collimator and the solid water phantom (with upstream surface‐mounted films) was varied. A 5‐pulse delivery was used. (a) Average PVDR with distance. (b) Average FWHM of peaks. (c) Average peak doses. Markers and error bars represent the mean value and standard deviation respectively of values obtained from the films via three symmetric single‐pixel line dosimetric profiles obtained with a 60^∘^ offset from one another whilst intercepting the centre of adjacent peaks, and the central axis.

As the solid water phantom was displaced farther from the collimator exit, the PVDR measured at the front surface decreased from 32 at the surface to 16 at ∼10 mm distance, and to 6 at ∼20 mm distance (Figure 6a). Meanwhile, the peak size increased (Figure 6b), with the average peak dose reducing exponentially ($R^{2}>0.99$) to ∼57% of the surface dose (29.5 to 16.9 Gy) at 10 mm distance, and to ∼33% (29.5 to 9.6 Gy) at 20 mm distance (Figure 6c).

### 4.2 Mouse phantom film measurements with eSFRT

**FIGURE 7:**
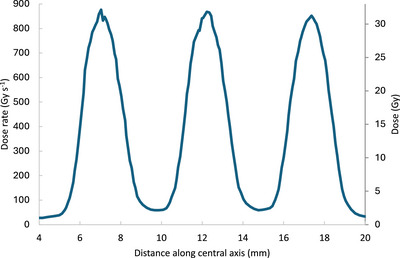
EBT‐XD film beam profile measurement of doses and dose rates at the surface of the mouse phantom (Figure 4) for an 11‐pulse irradiation using the 7‐hole electron insert (Figure 1, panel d).

Film measurements at the surface of the mouse phantom (Figure 4) showed that ultrahigh (FLASH) dose rates > 850 Gy s^−1^ were achievable (Figure 7). Here, the PVDR was also >14, with maximal surface peak dose rates within 3% of one another, and FLASH dose rates in surface valley regions between peaks of ∼60 Gy s^−1^ (identical film measurements with other collimators are indicated in Figure S1).

### 4.3 Preclinical experiments

Tumor growth varied significantly across treatment groups (Figure 8a, and Figure S5). The control group showed continuous tumor progression, whilst irradiation with eSFRT, with peak dose of 20 Gy, a valley dose of 1.4 Gy and an average dose of 6.7 Gy, at a conventional (< 0.1 Gy/s) dose rate (eSFRT‐CONV 20 Gy) resulted in modest growth inhibition. In contrast, both eSFRT‐FLASH groups demonstrated improved tumor control, with the eSFRT‐FLASH 30 Gy group (peak dose of 30 Gy, valley dose of 2.1 Gy and an average dose of 10 Gy, with local dose‐averaged dose rates at representative peak and valley voxels of 820 Gy/s and 60 Gy/s, respectively) exhibiting a more pronounced and sustained effect. No differences in tumor volume were observed within day 14. By day 21 (d21), the eSFRT‐FLASH 30 Gy group had significantly smaller tumors than the control (control vs. eSFRT‐FLASH 30, *p* = 0.027) and eSFRT‐CONV 20 Gy groups (eSFRT‐CONV 20 vs. eSFRT‐FLASH 30, *p* = 0.019). At day 30 (d30), the significant difference persisted only between the eSFRT‐CONV 20 Gy and the eSFRT‐FLASH 30 Gy groups (eSFRT‐CONV 20 vs. eSFRT‐FLASH 30, *p* = 0.021), while the difference between control and eSFRT‐FLASH 30 Gy was no longer statistically significant (*p* = 0.1). The tumor growth in the eSFRT‐FLASH 30 Gy group was comparable to that in the homogeneous eFLASH 10 Gy group, indicating similar therapeutic benefit from spatial dose fractionation.

The time to triple tumor volume (TTTV) was evaluated. TTTV varied substantially between treatment groups, reflecting differences in therapeutic efficacy (Figure 8b). In the control group, the median TTTV was 12.5 days, indicating rapid tumor progression and early endpoint attainment. Treatment with eSFRT‐CONV 20 Gy modestly extended TTTV to 17 days. A greater benefit was observed in the eSFRT‐FLASH 20 Gy group, with a median TTTV of 19 days. The eSFRT‐FLASH 30 Gy group showed a similar improvement, with a median TTTV of 25 days. Notably, the homogeneously treated tumors in the FLASH 10 Gy group also achieved a median TTTV of 25 days, with some mice surviving beyond 60 days, suggesting a more sustained suppression of tumor growth. Analysis of TTTV revealed that tumors in the FLASH 10 Gy group (*p* = 0.011), eSFRT‐FLASH 20 Gy group (*p* = 0.029), and eSFRT‐FLASH 30 Gy group (*p* = 0.036) reached triple their initial volume significantly later than those in the control group, with the largest delays observed for eSFRT‐FLASH 30 Gy. Additionally, TTTV was significantly prolonged in the eSFRT‐FLASH 30 Gy group compared with the eSFRT‐CONV 20 Gy group (*p* = 0.032).

Our findings suggest that eSFRT‐FLASH 30 Gy is effective at delaying tumor progression and providing prolonged tumor control comparable to the homogeneous eFLASH 10 Gy group. In addition, no significant differences in body weight as well as no skin damage were observed, indicating acceptable treatment tolerability (Figure 8c and Figure S6).

## 5 DISCUSSION

Our electron inserts enable a straightforward and flexible modulation of spatial fractionation parameters in the beamline. Reducing the CTC distances between collimator holes can help mitigate the off‐axis falloff of the incident Gaussian beam profile, thereby reducing disparities in PVDR between the 7‐ and 19‐hole inserts. However, when the CTC distances ≤ hole diameters, the dose peaks begin to merge and the resulting biological effects are expected to resemble the those from a homogeneous irradiation. Measurements of the additional collimators show that decreasing hole diameter along with reducing CTC distances allow for more peaks to cover the tumor. However, this approach also reduces the dose rate and PVDR with depth. Additionally, prolonged treatment times associated with this configuration may introduce motion‐induced blurring effects. It is also evident that increasing the distance between the irradiated sample and the collimator offers a practical approach for fine‐tuning spatial modulation parameters (e.g. PVDR, FWHM, dose rate), without the need to manufacture multiple collimators.

Our measurements of PVDR are similar to other static GRID collimation systems,^39^, ^40^, ^41^ with higher dose rates, but present a challenge for non‐superficial spatial modulation, as heterogeneity of dose quickly diminishes with depth.^29^, ^30^ Small Animal Radiation Research Platforms (SARRP) can improve accessibility and reduced the cost of MBRT with kV beams compared to synchrotrons, (with shortened delivery times and source‐target distances^42^, ^43^). While the peak doses and PVDRs are better maintained with depth, orthovoltage systems are also more limited when it comes to exploring the importance of ultrahigh dose rates for SFRT, as high power input may risk anode overheating without additional modifications to spot size, heat capacity, rotation rate, or employing scanned beams or parallel‐opposed configurations.^44^

While spatial modulation at a 20 Gy peak dose under conventional dose rates showed only modest improvement in tumor control relative to untreated controls, delivering the same peak dose at FLASH dose rates prolonged TTTV. This suggest that a quick delivery, mitigating motion‑induced blurring of dose peaks can be beneficial from a tumour response perspective. This potential benefit of delivering SFRT at FLASH dose rates requires further studies. Moreover, increasing peak dose to 30 Gy under FLASH dose rate resulted in more sustained and substantial tumor growth inhibition than the 20 Gy regimen, suggesting a dose‐dependent therapeutic benefit. Interestingly, the 30 Gy eSFRT‐FLASH regimen, despite delivering only a low (∼ 2 Gy) valley dose to most of the tumor (with an average dose of 10 Gy), was similarly effective in delaying tumor growth as the 10 Gy homogeneous eFLASH treatment (Figure 8). Our previous data demonstrated that higher homogenous irradiation doses further delay tumor growth with comparable tumor response between conventional and FLASH dose rates in the same animal model (Figure S7 and Table S2). However, homogeneous doses > 20 Gy often cause intolerable skin toxicity.^30^, ^45^ Spatial modulation offers a method to overcome these toxicity constraints, while potentially achieving improved tumor control. Notably, in all treatment groups in this study, including those with high SFRT peak dose, doses were well‐tolerated, with no significant weight loss or skin damage observed, highlighting the feasibility of dose escalation in SFRT. Together, our results suggest that the combined effects of spatial modulation, higher peak doses, and (potentially) FLASH dose rates contribute to an improved therapeutic response.

The apparent early underperformance of the eSFRT‐CONV 20 Gy group relative to controls may be explained by baseline tumor‐volume differences rather than treatment effects. This cohort exhibited larger initial tumor volumes (mean = 93.6 ± 10.8 mm^3^) compared to controls (mean = 72.3 ± 7.0 mm^3^), with a broader distribution. As tumor growth kinetics in this model scale with initial size, larger tumors tend to exhibit faster early progression, which can visually bias growth curves. Importantly, statistical analysis confirmed that these baseline differences were not significant (Tukey's multiple comparisons, adjusted *p* = 0.52 to > 0.9999), indicating that the observed trends reflect biological variability rather than systematic group differences.

Several limitations in our current study should be acknowledged. First, our electron‐based mini‐grid SFRT platform is constrained to superficial tumors at depths up to ∼5 mm, due to dose blurring of the peaks. Investigations beyond this depth will require the use of higher energy electron or photon beams.

Second, the presented tumor response study is limited by the restricted intratumoral spatial heterogeneity achievable with the current geometry. Tumor size at irradiation (mean ∼90 mm^3^, ∼3 mm thickness) constrained the number of peaks and valleys that could traverse the target (Figure 3), such that only a small number of peak‐valley transitions were present within the tumor volume. As a result, the degree of heterogeneity is substantially lower than that achieved in clinical LRT or MRT/MBRT systems, which employ multiple vertices or repeated high‐PVDR peak‐valley patterns across the target. This limitation has important implications for interpretation: instead of attributing the observed effects to the highly periodic spatial modulation characteristic of MRT/MBRT or dense Lattice approaches, the data should be interpreted as demonstrating the biological impact of clinically achievable electron‐based spatial modulation, where only limited peak‐valley structure is present within the tumour. Additionally, lateral scattering and penumbral spread resulted in partial overlap of peak regions with adjacent normal tissues, which may influence both tumor‐stroma interactions and normal tissue exposure. The biological impact of this peripheral exposure likely depends on spot size, spacing, and the dose rate delivered in both peak and valley regions.

Another limitation lies in the challenges of comparing homogenous and spatially modulated dose regimes.^46^ While we use average dose (defined here as the total dose delivered over a consistent enclosing area) as a comparative metric, this metric may not adequately reflect the biological response across modalities. More representative or biologically weighted measures are needed to accurately compare treatment outcomes and guide future SFRT treatment planning.

Despite these constraints, demonstrating measurable biological effects under these conservative modulation conditions provides a useful bridge between homogeneous electron irradiation and more strongly modulated SFRT approaches. Importantly, the platform enables systematic escalation of spatial heterogeneity through adjustment of collimator geometry, beam arrangement, and target size in future studies. As described above and in the Supplementary Material, additional collimators with smaller apertures and reduced spacing have been developed to further enhance intratumoral modulation in future studies.

Future work will focus on expanding our platform to accommodate deeper tumor targets by developing photon‐based collimation systems. In particular, the use of thick Cerrobend or tungsten blocks can provide sufficient photon attenuation for effective spatial modulation, allowing direct comparisons with our current electron mini‐grid SFRT inserts. Further optimization of SFRT beam geometry and placement are also required. Reducing CTC distances and hole diameters may enable more extensive tumor coverage but must be balanced against the risk of peak merging and compromised spatial modulation. In parallel, the biological impact of dose delivered to surrounding normal tissues, especially in peripheral peaks, warrants detailed evaluation, including its potential contribution to local or systemic immune responses. Modifying insert thickness would also alter valley doses, enabling the evaluation of valley dose as a metric for biological endpoints such as normal‐tissue toxicity, tumor control, and increased lifespan,^47^, ^48^ and to better mimic clinical SFRT scenarios.^19^

Lastly, the therapeutic potential of SFRT may be enhanced when used as a priming strategy in combination with immunotherapy and/or chemotherapy.^9^, ^49^ Ongoing studies will aim to characterize how spatial modulation and ultrahigh dose rates (FLASH) interact to shape immune activation, normal tissue sparing, and tumor response, ultimately informing optimal treatment strategies for future clinical translation.

## 6 CONCLUSIONS

We presented simple collimation systems for preclinical spatially fractionated electron radiotherapy, integrated into an established preclinical linac platform. Our 7‐ and 19‐hole brass inserts produced high PVDRs (35 and 31 respectively) and peak dose rates (ranging from  1.8 kGy s^−1^ to  850 Gy s^−1^). This platform is limited to investigating subcutaneous tumor response or treatment toxicity in superficial organs (e.g., skin) of mice, but its flexibility in dose rate and collimator arrangements make it an attractive complement to orthovoltage systems for preclinical SFRT studies.

In a subcutaneous tumor model, a 30 Gy peak dose (with a low 2.1 Gy valley dose, and a 10 Gy average dose) delivered at FLASH dose rates achieved tumor control comparable to a homogeneous 10 Gy FLASH regimen and significantly outperformed 20 Gy peak dose (1.4 Gy valley dose, and a 6.7 Gy average dose) SFRT delivered at a conventional dose rate. These results highlight the potential of combining spatial modulation, higher peak doses, and FLASH dose rates to enhance therapeutic response while maintaining dose tolerability.

This system enables flexible modulation of beam geometry and dose rate, providing a robust framework to support the investigation of SFRT across a wide range of parameters, and guide future clinical translation.

## CONFLICT OF INTEREST STATEMENT

The authors have no relevant conflicts of interest to disclose.

## Supporting information

Supporting Information
