# Supplementary material for: A preclinical setup for spatially fractionated radiation therapy with electrons
Source: Med Phys. 2026 Apr 24;53:e70448. doi: 10.1002/mp.70448 (PMC13107147; doi:10.1002/mp.70448)
Supplement: Supplementary file 1 — Supporting Information [file MP-53-0-s001.docx]

# Supplementary material

A

B

C

**Figure S1**: Electron 7-hole insert EBT-XD film irradiations at various depth in solid water where 0 mm indicates the surface. A 5-pulse delivery was used for all inserts with different hole diameters (∅) [0.65-1.3 mm], and centre-to-centre distances (ctc) [1.625-5 mm]. A gun current of 7.34 A was used for 0.65 mm ∅ irradiations, whilst 7.3 A was used for 1.00 mm ∅ and 1.30 mm ∅ irradiations. An enhanced dpi of 600 was used to analyse films, here: A) Average peak-to-valley dose ratio (PVDR) with depth B) Average full width at half maximum (FWHM) of peaks C) Average relative peak doses. Markers and error bars represent the mean value and standard deviation respectively of values obtained from the films via three symmetric single-pixel line dosimetric profiles obtained with a 60^∘^ offset from one another whilst intercepting the centre of adjacent peaks, and the central axis.

**Table S1**: Monte Carlo methods used in simulations following recommendations from TG 268 [1]

| **Item name** | **Description** | **References** |
| --- | --- | --- |
| Code, version/release date | EGSnrc v4 2025-1-15 | [2] |
| Validation | Faddegon et al. 2009,  Shiekh-Bagheri et al 2000,  Failing et al. 2022, | [3],[4],[5] |
| Timing | Intel Platinum 8628, Cascade Lake, 2.90GHz CPU with 48CPU cores, and 380GB RAM | [6] |
| Source description | egs_chamber: Monoenergetic 6 MeV electrons, Gaussian with sigma=0.0951 cm  dosxyznrc: phase space from egs_chamber output |  |
| Cross-sections | Photon cross sections = xcom,  Pair angular sampling = Simple,  Brems cross section = NRC,  Brems angular sampling = KM |  |
| Transport parameters | AE=ECUT=0.521 MeV  AP=PCUT=0.01MeV,  ESTEPE=0.25,  SMAX=1e10,  XIMAX=0.5,  Boundary crossing=exact,  Skin depth for BCA=3,  Electron-step algorithm=EGSnrc |  |
| VRT and/or AEIT | Not used | [7] |
| Scored quantities | egs_chamber: Phase space at solid water surface, dosxyznrc: doses in water voxels of size – 0.26x0.26x1 mm^3^ [similar to 96 dpi], depth shown at centre of voxel |  |
| Histories/Statistical uncertainty | 1e9/Shown as error bars on figures |  |
| Statistical methods | History-by-history | [8] |
| Postprocessing | Performed in python:   - PVDR = Average peak/Average valley [those between peaks] - FWHM = Average width between 50% points in graph normalised to maximum - Maximum dose in spot = Normalised to value at 0.5 mm depth |  |


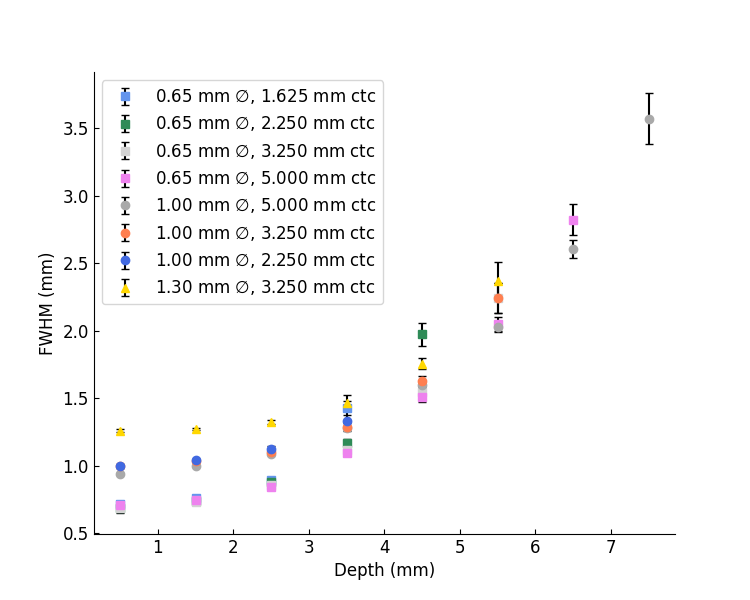

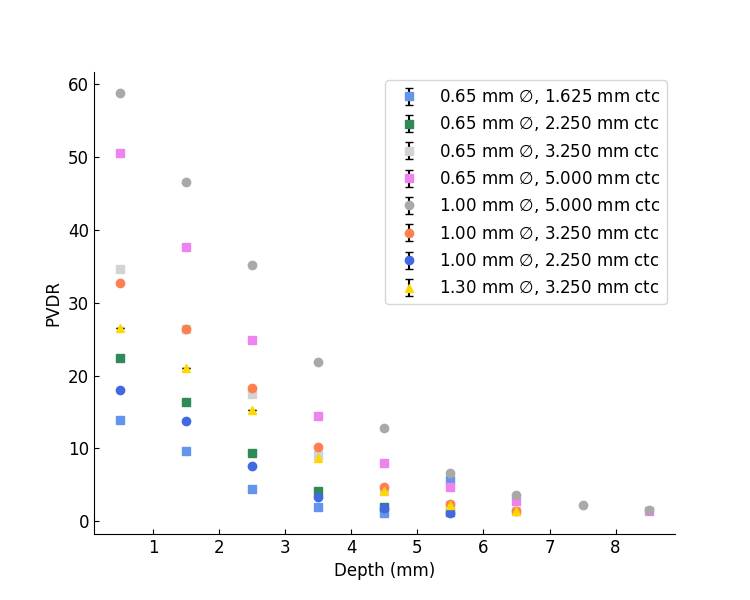


B

A

**
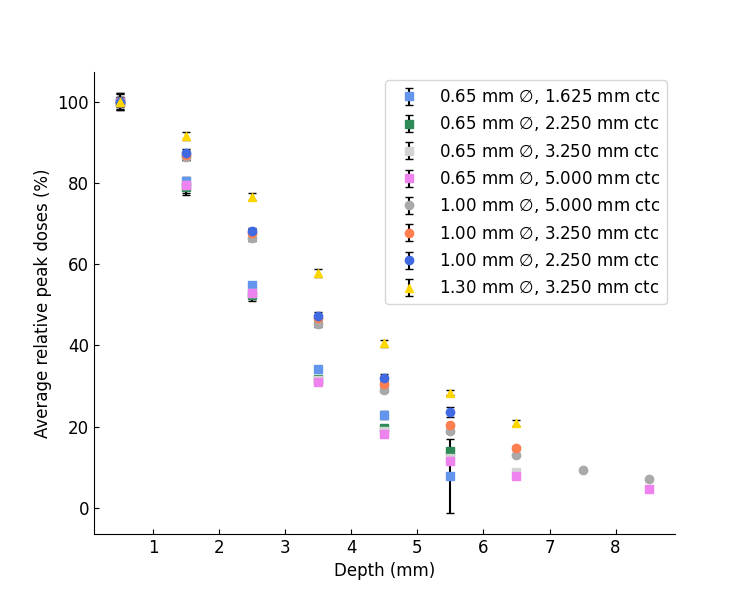
**

C

**Figure S2**: Electron 7-hole insert EGSnrc simulations (described in Table S1) at various depth in solid water where 0 mm indicates the surface. A 5-pulse delivery was used for all inserts with different hole diameters (∅) [0.65-1.3 mm], and centre-to-centre distances (ctc) [1.625-5 mm], here: A) Average peak-to-valley dose ratio (PVDR) with depth B) Average full width at half maximum (FWHM) of peaks C) Average relative peak doses. Markers and error bars represent the mean value and standard deviation respectively of values obtained from the films via one symmetric single-pixel line dosimetric profile intercepting the centre of adjacent peaks, and the central axis.

B

A

C

**Figure S3**: Electron 7-hole insert EBT-XD film irradiations at the surface of the mouse phantom (Figure 4). An 8-pulse delivery was used for all inserts with different hole diameters (∅) [0.65-1.3 mm], and centre-to-centre distances (ctc) [1.625-5 mm]. A gun current of 7.3 A was used for all irradiations. An enhanced dpi of 600 was used to analyse films, here: A) Average peak-to-valley dose ratio (PVDR) with depth B) Average full width at half maximum (FWHM) of peaks C) Peak doses. Markers and error bars represent the mean value and standard deviation respectively of values obtained from the films via three symmetric single-pixel line dosimetric profiles obtained with a 60^∘^ offset from one another whilst intercepting the centre of adjacent peaks, and the central axis.


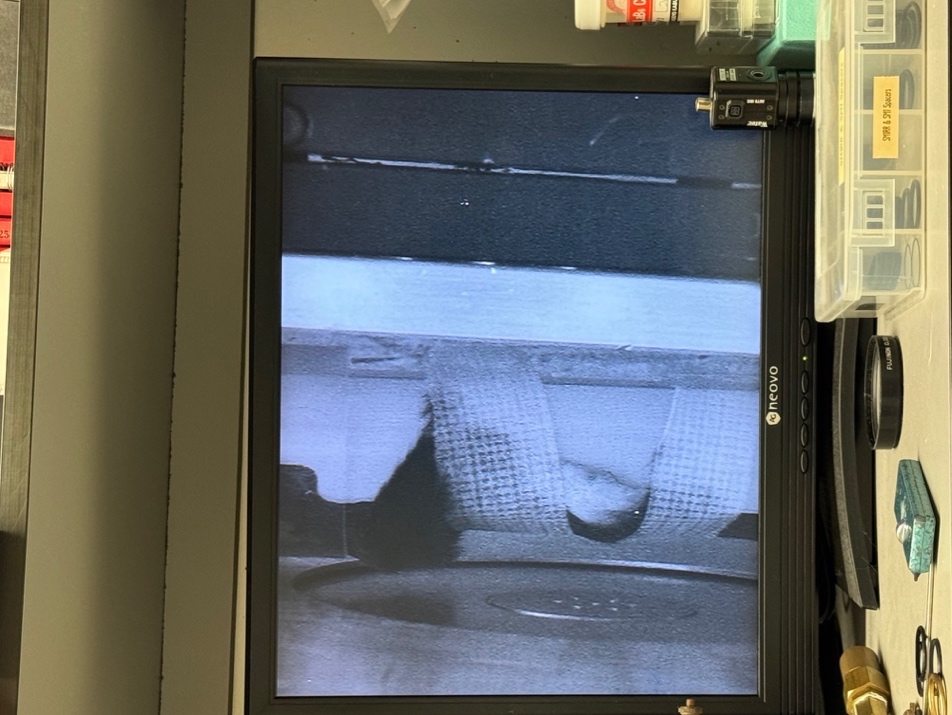

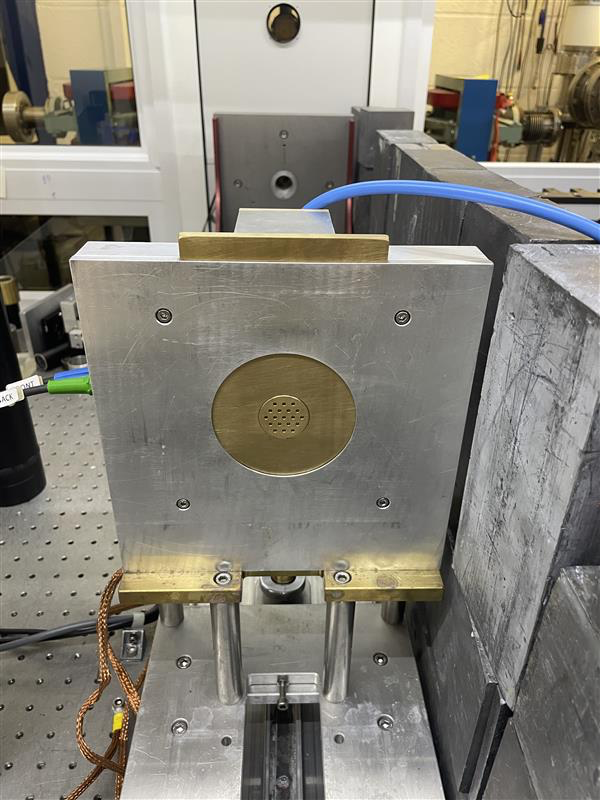

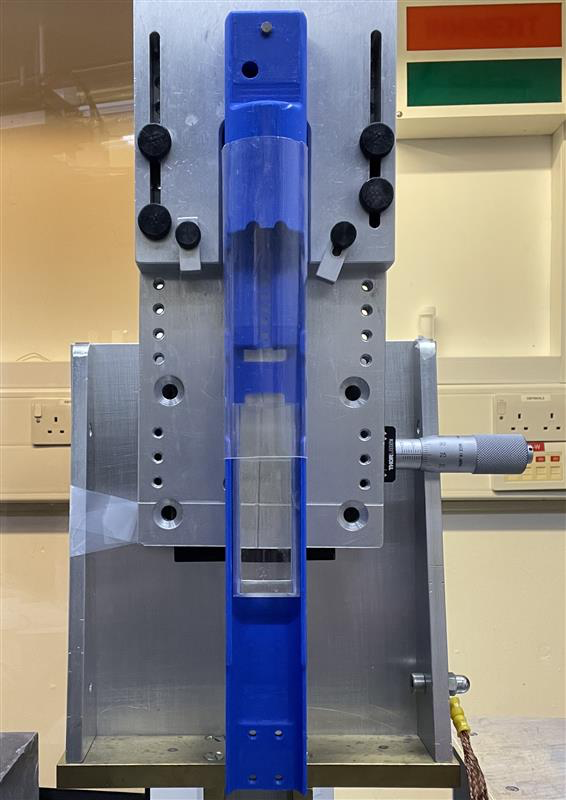

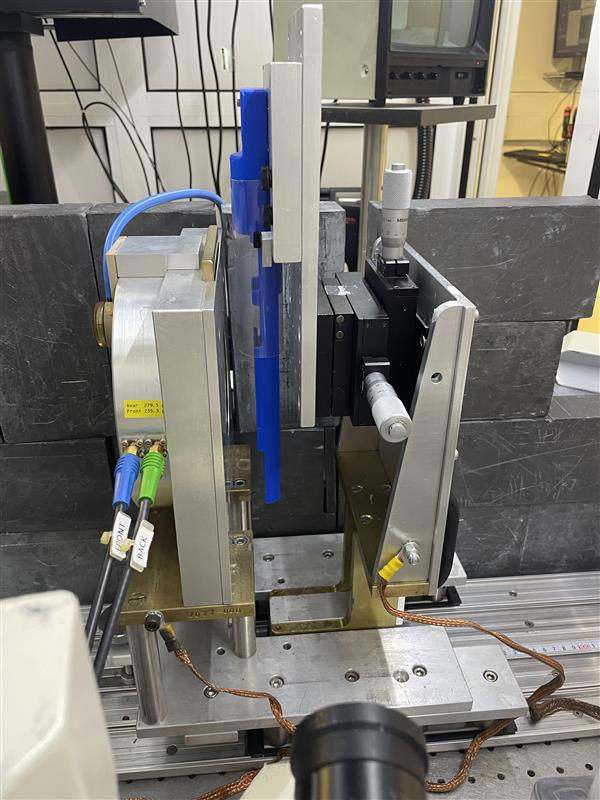


**A**

**B**

**C**

Ti scattering foil

Mouse phantom front curved surface

Brass collimator plate

Energy monitor

**D**

19-hole brass insert

Mouse positioned upright within custom cradle

**Figure S4:** (A) Side view of preclinical mouse irradiation platform beamline setup (B) Downstream view of mouse phantom within the mouse cradle (C) Upstream view of brass collimator plate and 19-hole insert. (D) Photograph of the irradiation setup illustrating animal positioning and beam alignment. Mice were placed upright in a custom cradle with the flank tumour positioned directly over the central aperture of the SFRT collimator. In this orientation, the electron beam traverses only skin and subcutaneous tissues of the flank, while abdominal organs—including the intestine—remain outside the field. This configuration ensures accurate targeting of the tumour and prevents inadvertent irradiation of deeper tissues.

**Figure S5:** Initial tumor volume and tumor growth curves for individual mice in each treatment group. Mice were irradiated at day 0. For the dot plot, each dot represents an individual mouse, with horizontal bars indicating the group mean ± SEM. For the line graphs, each line represents a single mouse, illustrating the variation in tumor growth trajectories over time.

**Figure S6**: Dot plot of percentage body-weight change at day 9 post-irradiation for all treatment groups. Each point represents an individual mouse, with horizontal bars indicating the group mean ± SEM.

**
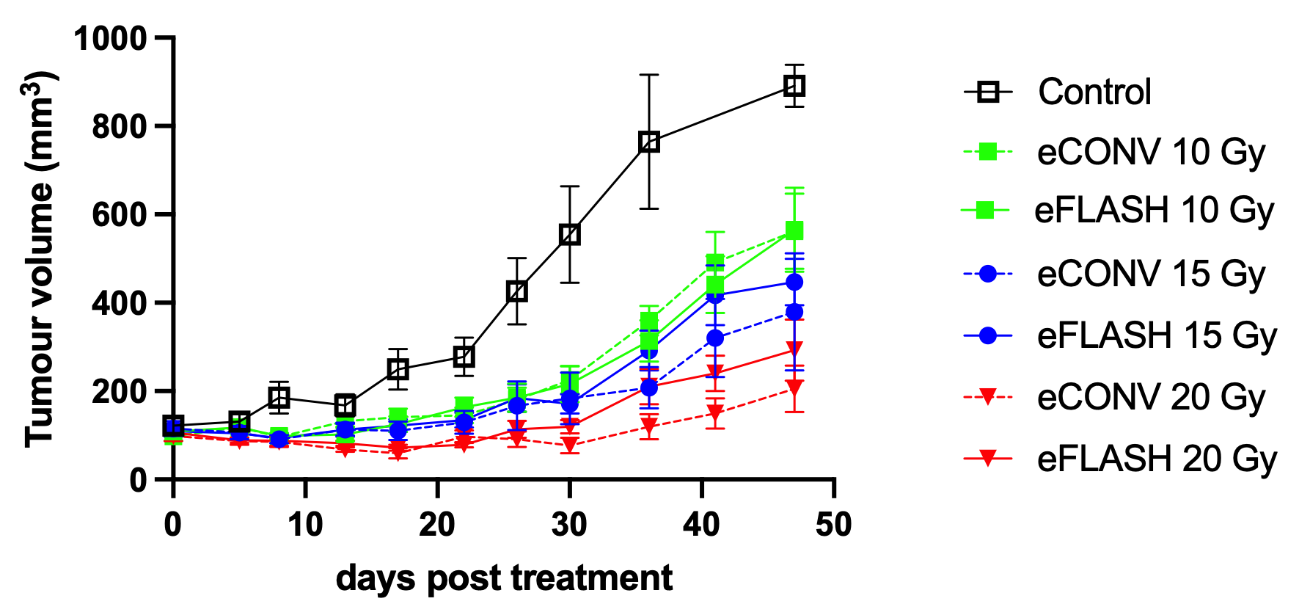
**

**Figure S7**: Tumor growth curve from homogenous electron irradiations for mice with subcutaneous UPPL1541 tumors. Initial tumor volume at day 0 was 108 ± 28.93 mm³ (mean ± SD). Sample sizes were n = 5 for the control group and n = 6 for all other treatment groups.

**Table S2**: Šidák’s multiple comparisons p value tests of irradiated groups shown in Figure S6. Here markers indicate levels with: ns p≥0.05, * p<0.05, *** p<0.001, **** p<0.0001

|  | **Control** | **eCONV 10 Gy** | **eCONV 15 Gy** | **eCONV 20 Gy** | **eFLASH 10 Gy** | **eFLASH 15 Gy** | **eFLASH 20 Gy** |
| --- | --- | --- | --- | --- | --- | --- | --- |
| **Control** |  | **** | **** | **** | **** | **** | **** |
| **eCONV 10 Gy** |  |  | **ns** | **** | **ns** | **ns** | **** |
| **eCONV 15 Gy** |  |  |  | **ns** | **ns** | **ns** | **ns** |
| **eCONV 20 Gy** |  |  |  |  | *** | * | **ns** |
| **eFLASH 10 Gy** |  |  |  |  |  | **ns** | *** |
| **eFLASH 15 Gy** |  |  |  |  |  |  | * |
| **eFLASH 20 Gy** |  |  |  |  |  |  |  |

# References

1. Sechopoulos, I., et al., *RECORDS: improved Reporting of montE CarlO RaDiation transport Studies: Report of the AAPM Research Committee Task Group 268.* Medical Physics, 2018. **45**(1): p. e1-e5.

2. National Research Council of Canada. Metrology Research Centre. Ionizing Radiation, S., *EGSnrc: software for Monte Carlo simulation of ionizing radiation*. 2021, National Research Council of Canada.

3. Faddegon, B.A., et al., *The accuracy of EGSnrc, Geant4 and PENELOPE Monte Carlo systems for the simulation of electron scatter in external beam radiotherapy.* Physics in Medicine and Biology, 2009. **54**(20): p. 6151-6163.

4. Sheikh-Bagheri, D., et al., *Comparison of measured and Monte Carlo calculated dose distributions from the NRC linac.* Medical Physics, 2000. **27**(10): p. 2256-2266.

5. Failing, T., et al., *Enhancement of the EGSnrc code egs_chamber for fast fluence calculations of charged particles.* Z Med Phys, 2022. **32**(4): p. 417-427.

6. Richards, A., *University of Oxford Advanced Research Computing.* 2015.

7. Wulff, J., K. Zink, and I. Kawrakow, *Efficiency improvements for ion chamber calculations in high energy photon beams.* Med Phys, 2008. **35**(4): p. 1328-36.

8. Walters, B.R.B., I. Kawrakow, and D.W.O. Rogers, *History by history statistical estimators in the BEAM code system.* Medical Physics, 2002. **29**(12): p. 2745-2752.
